# Dysphagia and esophageal dysfunction due to dystrophin deficient muscular dystrophy in a male Spanish water spaniel

**DOI:** 10.1080/01652176.2018.1435939

**Published:** 2018-02-20

**Authors:** Brigitte B. McAtee, Johanna C. Heseltine, Ling T. Guo, Michael D. Willard, G. Diane Shelton

**Affiliations:** a Department of Small Animal Clinical Sciences, Texas A&M University, College Station, TX, USA; b Comparative Neuromuscular Laboratory, University of California San Diego, La Jolla, CA, USA

**Keywords:** Dog, canine, muscular dystrophy, dysphagia, dystrophin-deficient, regurgitation

**Abbreviations**
dMHC: Developmental myosin heavy chain
DD-MD: Dystrophin deficient muscular dystrophy
H&E: Haemotoxylin and Eosin
IB: Immunoblotting
IF: Immunofluorescent
MD: Muscular Dystrophy
RI: Reference Interval

An 8-month-old, 16.0 kg, intact male Spanish water spaniel presented to the Texas A&M University, Veterinary Medical Teaching Hospital for evaluation of acute onset of hypersalivation, inappetence, and regurgitation one week prior to presentation. The dog was purchased from Spain and transported to the United States at three months of age. The parents and siblings of this dog were reportedly healthy. However, additional medical information regarding these dogs was not available. Prior to admission at the veterinary teaching hospital, diagnostic findings included radiographic dilation of the cervical and thoracic esophagus with gas plus mild gaseous distension of the stomach and intestines. Because of the radiographic esophageal abnormalities, an adrenocorticotropic hormone (ACTH) stimulation test was performed on the suspicion of Addison's disease and was within normal limits (baseline cortisol 129.7 nmol/L; reference interval (RI) 27.6–138.0 nmol/L, one-hour post-ACTH stimulation cortisol 380.7 nmol/L using an unknown amount of cosyntropin; RI 220.7–469.0 nmol/L). Various supportive treatments for acute regurgitation and esophagitis did not produce clinical improvement, and the dog was referred for additional diagnostics. Upon presentation to Texas A&M University, physical examination documented a body condition score (BCS) of 5/9 with no evidence of muscle atrophy, but hypersalivation with frequent swallowing attempts was noted. Neurological examination was normal. The alanine aminotransferase activity was moderately increased (650 U/L; RI 10–130 U/L) and creatine kinase (CK) activity was elevated (2846 U/L; RI 68–400); otherwise the CBC, serum chemistry panel, and urinalysis had no clinically important abnormalities. Three-view thoracic radiographs revealed moderate esophageal dilation and a mild, diffuse bronchial and interstitial lung pattern. Findings on abdominal ultrasound included a small volume of peritoneal effusion, atypical enlarged vessels in the abdomen suspected to be portosystemic shunts, and small urinary bladder calculi.

The acetylcholine receptor antibody titer was within the reference range (0.15 nmol/L; RI < 0.6 nmol/L). Total serum thyroxine concentration was mildly decreased (17.5 nmol/L; RI 21.9–46.3 nmol/L), but free T4 and TSH concentrations were normal (15.5 pmol/L; RI 7.7–47.6 pmol/L and 0.1 ug/L; RI 0.05–0.42 ug/L, respectively). Cobalamin and folate levels were within normal limits at >740 pmol/L [RI 185–670 pmol/L] and 20.9 nmol/L [RI 17.5–55.3 nmol/L], respectively. The dog was fed canned gastrointestinal dog food^1.^ shaped into ‘meatball-sized’ servings that he received in a Bailey Chair.^2.^ The dog was kept upright for 20 min after eating. During eating, swallowing was difficult and numerous attempts to swallow were preceded by apparent passing of food through the esophagus. No further episodes of regurgitation were noted, and appetite returned to normal. Hypersalivation between feedings was still noted. Cisapride (0.5 mg/kg, PO, q 8 h), omeprazole (1.3 mg/kg, PO, q 24 h), ondansetron (0.5 mg/kg, PO, q 12 h), and S-adenosylmethionine/silybin (225 mg/24 mg, PO, q 24 h), plus continued Bailey Chair^2^ feedings of canned gastrointestinal diet dog food^1^ shaped into ‘meatball-sized’ servings did not help. At 9 days after discharge, the dog was lethargic and was regurgitating after every meal for the previous two days. A slightly elevated rectal temperature (102.4 °F, 39.1 °C) and exaggerated swallowing attempts when the ventral cervical region was palpated were noted. Repeat thoracic radiographs (Figure 1) revealed that the megaesophagus was more severe and generalized compared to previous radiographs from the referring veterinarian, with no evidence of aspiration pneumonia. Contrast-enhanced fluoroscopic evaluation of the esophagus revealed segmental megaesophagus cranial to the severely narrowed area of the mid to caudal esophagus and hypomotility with near complete obstruction of the caudal esophagus (Figure 2). No food or liquid barium bypassed this obstruction.

**Figure 1.**
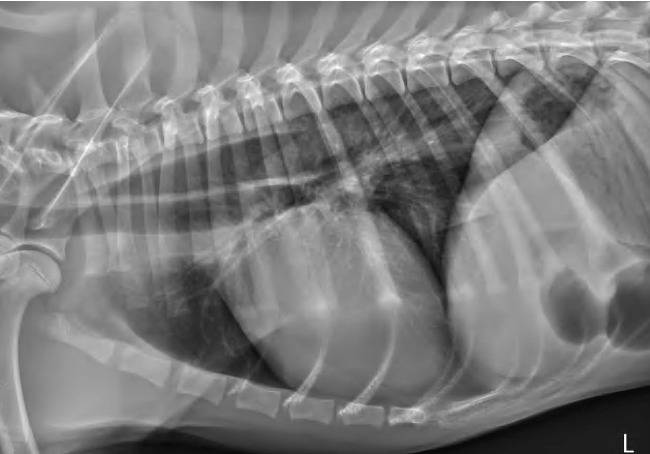
Left lateral radiograph of the thorax revealing a gas-distended esophagus that is more noticeable in the cranial thorax.

**Figure 2.**
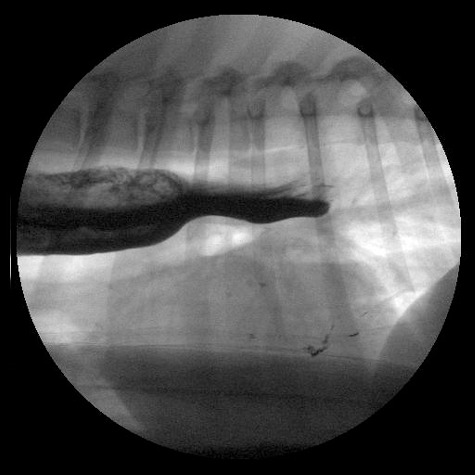
Lateral fluoroscopic projection of the intra-thoracic esophagus. This image reveals segmental megaesophagus and hypomotility, with near complete obstruction of the caudal esophagus.

The following day, the esophagus was evaluated with a computed-tomography (CT) scan and esophagoscopy. The CT scan revealed circumferential thickening of the caudal esophageal wall from the level of the tracheal carina to the gastroesophageal junction (Figure 3). Cranial to the tracheal carina, the esophageal wall thickness was normal. However, there was mild segmental dilation. Other incidental findings on the CT scan included a diaphragmatic hernia and bifid seventh sternebral segment and xiphoid.

**Figure 3.**
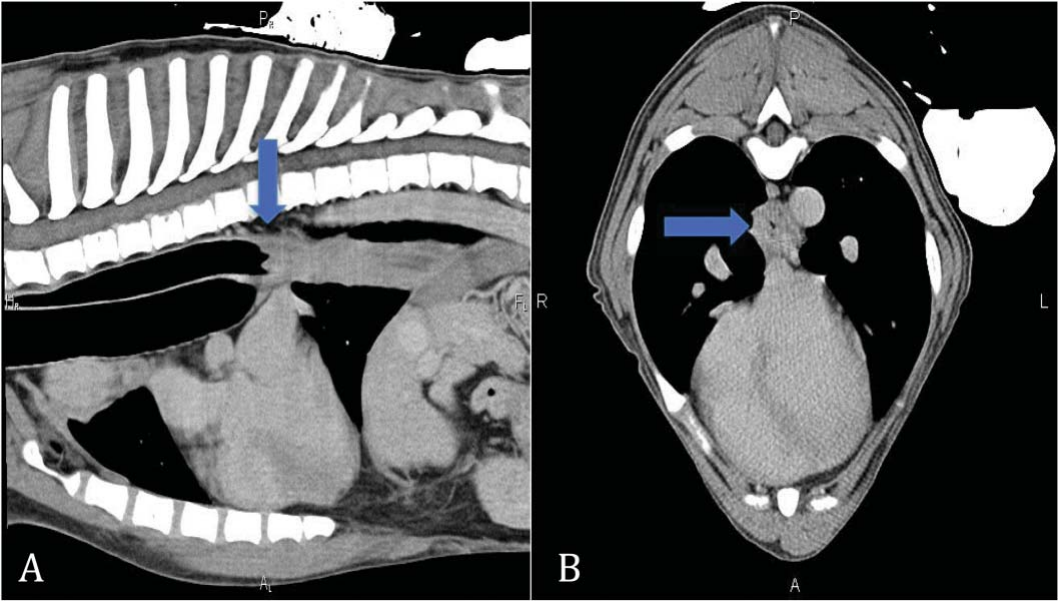
Computed tomography sagittal reconstruction (A) and transverse (B) images of patient's thorax. The caudal esophageal wall is circumferentially thickened and mildly undulant along its luminal surface from the level of the tracheal carina caudally to the gastroesophageal junction indicating the focal area of muscular dystrophy. Arrows identify the start of the thickening in the caudal esophagus on both images.

Esophagoscopy revealed a circumferentially thickened area with numerous folds that resembled an extramural obstruction. The thickened area was about four centimeters orad to the lower esophageal sphincter (Figure 4). The lower esophageal sphincter abnormally opened and closed with breathing (Figure 4). Endoscopic esophageal biopsies of the thickened, narrow area were taken with flexible biopsy forceps. The esophageal mucosa appeared histologically normal with H&E staining. A feeding tube to provide nutritional support was declined, and the owners elected for euthanasia after two days of hospitalized care.

**Figure 4.**
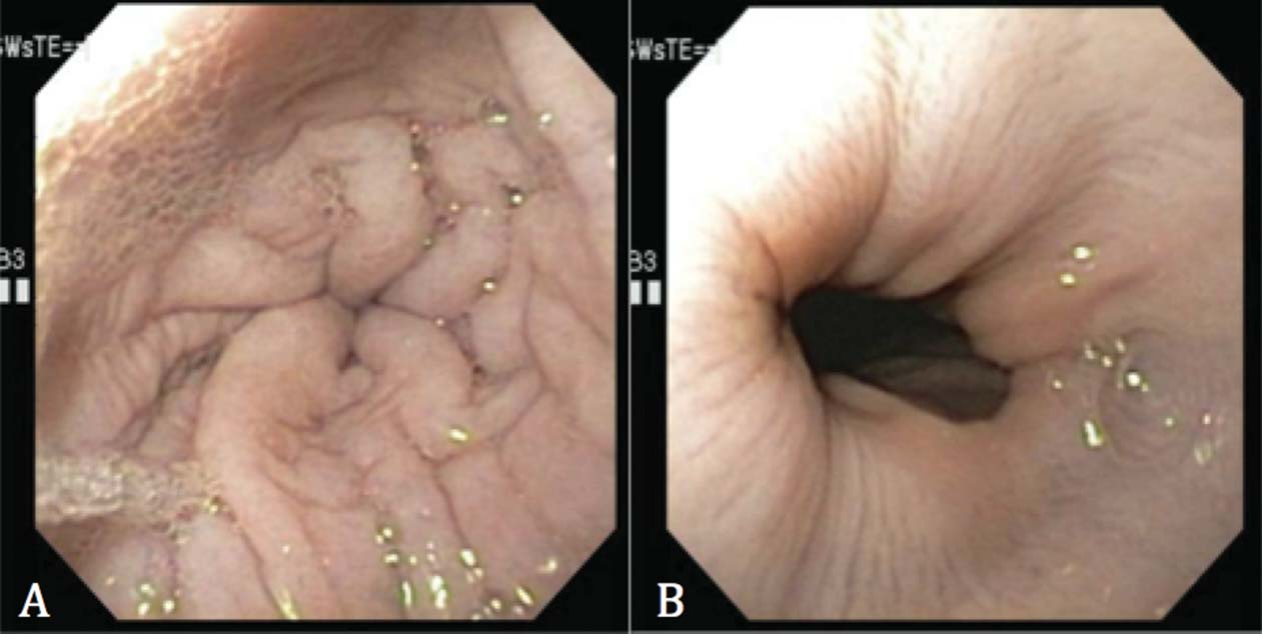
Esophagoscopy images of the caudal esophagus (A) and lower esophageal sphincter (B). The esophageal wall in A appears circumferentially thickened and mildly undulant along its luminal surface, similar to what would be expected of an extramural compressive lesion. The lower esophageal sphincter (B) opened and closed with respirations.

Necropsy examination revealed moderate, multifocal, and chronic myofiber degeneration of the esophageal muscles. Similar findings were identified in the diaphragmatic muscles and cardiac muscles. Other findings on the necropsy included bronchopneumonia and an extra-hepatic shunt. Final diagnosis was a chronic degenerative myopathy involving striated muscles (esophageal, cardiac, diaphragm). Frozen specimens from several unnamed skeletal muscles were submitted to the Comparative Neuromuscular Laboratory.^3.^ Cryosections from a representative muscle showed large clusters of degenerating and regenerating fibers consistent with a non-inflammatory, most likely dystrophic, myopathy (Figure 5).

**Figure 5.**
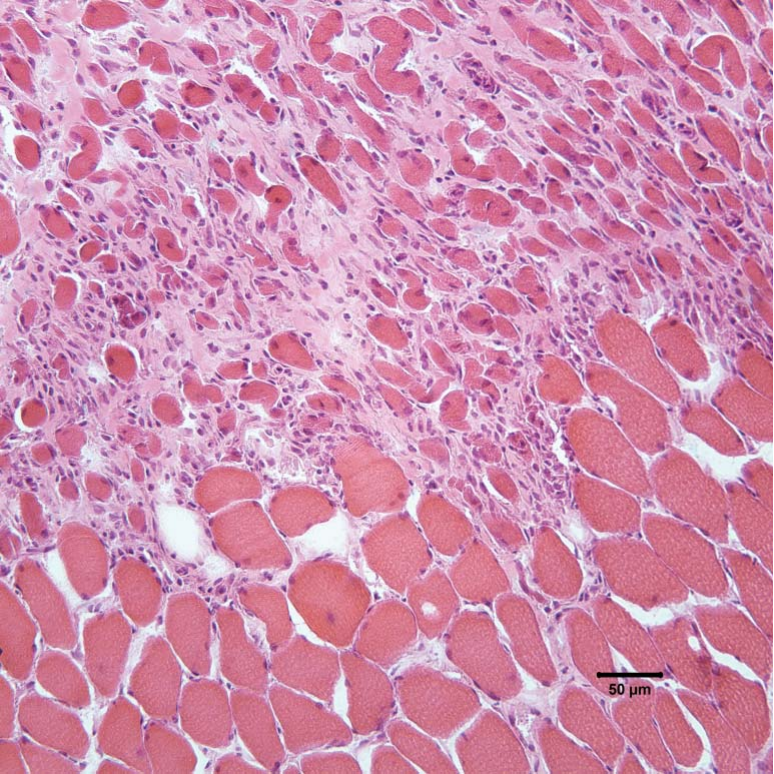
H&E stained frozen section of an unidentified skeletal muscle showing a large cluster of degenerating myofibers (upper half of image) adjacent to myofibers of more normal size and shape. H&E stain.

To further characterize a form of MD, IF staining was performed on additional cryosections for localization of several dystrophy-associated proteins using monoclonal and polyclonal antibodies against the rod and carboxy-terminus of dystrophin, utrophin, laminin α2, dysferlin, the dystrophin associated glycoproteins (α, β, and γ-sarcoglycans, β-dystroglycan), caveolin 3, spectrin, lamin AC, and dMHC using previously published methods (Vieira et al. 2015). Compared to control muscle, staining was appropriate for the rod domain of dystrophin but was absent for the carboxy-terminus. A large group of regenerating fibers was identified using the antibody against dMHC (Figure 6(a)). IB was performed using previously published methods (Vieira et al. 2015). While the IF staining pattern using the antibody against the rod domain of dystrophin was appropriate, the amount of protein detected by IB was decreased. A fragment was not detected using the antibody against the carboxy terminus of dystrophin. Loading controls for both the dystrophic dog and control dog were similar as determined by staining for β-actin (Figure 6(b)). A dystrophinopathy was confirmed with IF and IB analysis.

**Figure 6.**
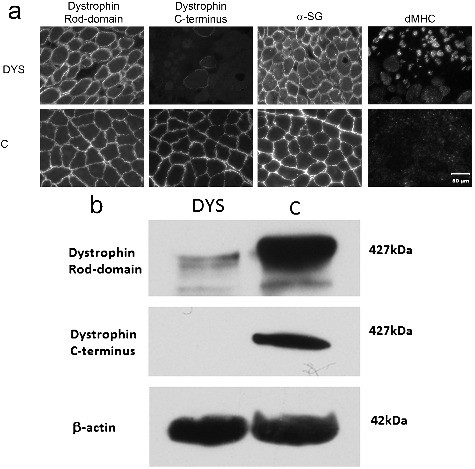
(a). Immunofluorescence staining of cryosections from an unidentified skeletal muscle of the dystrophic (DYS) dog of this report and archived control (C) limb muscle. Stainings shown used antibodies against the rod and carboxy terminus of dystrophin, α- sarcoglycan (SG) and developmental myosin heavy chain (dMHC) for regenerating fibers. Bar in lower right image = 50 μm for all images. (b). Immunoblot stained for detection of the rod domain and carboxy-terminus of dystrophin from the dystrophic dog (DYS) and archived control (C) muscle. An antibody against β-actin was used as a loading control. Note the decreased amount of the protein fragment with a normal molecular size using the antibody against the rod domain and the absence of staining with the antibody against the carboxy terminus.

This report highlights a unique presentation of MD with regurgitation as the primary presenting clinical sign without obvious gait abnormalities or other neurological signs.

Dysphagia and esophageal dysfunction in this dog are likely explained by MD affecting the pharyngeal and esophageal musculature, in addition to subclinical limb muscle involvement.

Muscular dystrophies are a diverse group of inherited, degenerative myopathies that are typically non-inflammatory (Shelton and Cardinet 1987). Similar to human medicine, most cases of canine MD are caused by DD-MD, an X-linked, hereditary condition (Valentine et al. 1988; McGreevy et al. 2015). Canine patients with DD-MD serve as a model for Duchenne muscular dystrophy in humans. However, other types of MD, including limb girdle MD caused by sarcoglycan deficiency, have also been described in dogs (Deitz et al. 2008; Munday et al. 2015; Cox et al. 2017). Staining for α-sarcoglycan in this case was similar to control muscle (Figure 6(a)).

DD-MD has been described in several breeds including golden retrievers (Cooper et al. 1988), lurchers (Giannasi et al. 2015), Labrador retrievers (Vieira et al. 2015), Alaskan malamutes (Ito et al. 2011), and others. Gene mutations for DD-MD have been identified in a few breeds, including Cavalier King Charles spaniels, rottweilers, and golden retrievers (McGreevy et al. 2015). Sarcoglycan deficient MD is a less commonly reported form of MD, but has been described in Boston terriers with the mutation recently identified in the gene encoding δ-sarcoglycan (Shelton and Engvall 2005; Cox et al. 2017), and in a Doberman pinscher with suspected spontaneous mutation in a sarcoglycan gene (Munday et al. 2015). Genetic testing is necessary to confirm a mutation in MD, but was not pursued in this case. MD has not been described in the Spanish water spaniel. Limited medical history was available for the relatives of this dog; however, the breeder reported no abnormalities in this familial line. Neither a breed associated inherited dystrophy nor a spontaneous mutation can be ruled out.

Laboratory evaluations in cases of MD often include an elevated CK activity, which was noted in the dog of this report. It is important to note that this dog did not have obvious neuromuscular weakness or appreciable muscle atrophy. Electrodiagnostics were not performed. However, electromyography in dogs with MD shows bizarre high-frequency discharges and/or spontaneous activity in various muscle groups in the form of fibrillation potentials and positive sharp waves (Deitz et al. 2008, Dewey and Talarico 2016). Clinical signs of canine MD typically appear around two months of age and are characterized by a shuffling or stiff gait with shortened stride, difficulty eating, hypertrophy of tongue, and muscle atrophy (Shelton and Engvall 2005), with death typically occurring before three years of age. However, a large family of Labrador retrievers has been described with DD-MD and a very mild clinical phenotype (Vieira et al. 2015). Such variation in clinical severity has also been described in a colony of Irish terrier dogs with X-linked muscular dystrophy (XLMD) (Kornegay 2017).

The dog in this report had a unique esophageal lesion (Figure 4) with megaesophagus cranial to this lesion (Figure 3). Megaesophagus has been described with canine MD and was noted on 34.2% of thoracic radiographs of golden retrievers with MD in one study (Bedu et al. 2012). Another study (Gerger et al. 2010) showed that the esophagus and stomach were the most affected aspects of the digestive tract in golden retriever dogs with MD. However, the described focal circumferential thickening of the esophagus causing complete obstruction noted in this case has not been observed in other dogs with MD. One case report (Giannasi et al. 2015) described fluoroscopic evaluation of a dog with DD-MD, which revealed narrowing of the esophagus immediately caudal to the heart base causing passage of small amounts of food. However, the esophagus was normal on endoscopic evaluation. Diffuse thickening of the muscular wall of the esophagus has been described in canine XLMD (Valentine et al. 1990). However, these dogs did not have focal, circumferential thickening of the esophagus causing an obstruction.

The dog in this report also had a diaphragmatic hernia identified on the CT scan, which has been associated with MD (Lessa et al. 2014). The dog in this current report also had evidence of other congenital abnormalities, including congenital portosystemic shunts found on necropsy, and bifid seventh sternebral segment and xiphoid identified on the CT scan. While these findings are interesting, they have not been reported as being associated with congenital MD and are most likely epiphenomena.

The overall prognosis of DD-MD is poor, due to complications such as chronic dysphagia and regurgitation with subsequent aspiration pneumonia, and in many cases cardiomyopathy. There are no specific proven therapies currently available, and additional research is necessary to identify effective treatment options. Correct diagnosis, identification of pathologic genetic variants and development of diagnostic genetic tests may provide the most efficient way to rid breeding lines of MD. This case report should heighten the clinical awareness that generalized neuromuscular diseases can present with focal clinical signs referable to the pharyngeal or esophageal musculature.

## Notes

1. Purina ProPlan Veterinary Diets EN Gastroenteric®; Nestle Purina PetCare Company, St. Louis, MO.

2. Bailey Chair for upright feeding; https://www.baileychairs4dogs.com/

3. Comparative Neuromuscular Laboratory; School of Medicine, University of California San Diego, La Jolla, CA, USA. http://vetneuromuscular.ucsd.edu
